# Complementary Approaches to Retinal Health Focusing on Diabetic Retinopathy

**DOI:** 10.3390/cells12232699

**Published:** 2023-11-24

**Authors:** Tibor Rák, Andrea Kovács-Valasek, Etelka Pöstyéni, Adrienne Csutak, Róbert Gábriel

**Affiliations:** 1 Department of Ophthalmology, Clinical Centre, Medical School, University of Pécs, Rákóczi út 2., 7623 Pécs, Hungary; rak.tibor@pte.hu (T.R.);; 2 Department of Neurobiology, University of Pécs, Ifjúság útja 6, 7624 Pécs, Hungary; 3 János Szentágothai Research Centre, University of Pécs, Ifjúság útja 20, 7624 Pécs, Hungary

**Keywords:** diabetic retinopathy, NFκB, complementary and alternative medicines

## Abstract

Diabetes mellitus affects carbohydrate homeostasis but also influences fat and protein metabolism. Due to ophthalmic complications, it is a leading cause of blindness worldwide. The molecular pathology reveals that nuclear factor kappa B (NFκB) has a central role in the progression of diabetic retinopathy, sharing this signaling pathway with another major retinal disorder, glaucoma. Therefore, new therapeutic approaches can be elaborated to decelerate the ever-emerging “epidemics” of diabetic retinopathy and glaucoma targeting this critical node. In our review, we emphasize the role of an improvement of lifestyle in its prevention as well as the use of phytomedicals associated with evidence-based protocols. A balanced personalized therapy requires an integrative approach to be more successful for prevention and early treatment.

## 1. Introduction

### 1.1. Diabetes Mellitus and Its Health Impact on Numbers

The term diabetes mellitus (DM)—which literally means honey-sweet (“mellitus” in Latin) and copious urination (διαβήτης—ancient Greek for “pass over”)—refers to a symptom observed since ancient times: the appearance of sugar in the urine. It is an unstoppably advancing and incurable metabolic disease with a progressive, constantly worsening tendency, affecting mainly carbohydrate homeostasis, but also influences fat and protein metabolism [1,2]. Chronic hyperglycemia, sooner or later, results in long-term vascular damage, which leads to a series of complications, such as the dysfunction and failure of various organs (the heart, liver, stomach, kidney, muscles, peripheral nerves, etc.) [3]. By 2014, according to the Global Report on Diabetes, the disease was stated to cause heart attacks, stroke, renal failure, and lower limb amputation, which have deadly consequences. According to the International Diabetes Federation, the number of patients has quadrupled since 1980, so the number of patients increased to 422 million. In 2012, DM alone caused a 1.5 million patient mortality rate while its complications devitalized another 2.2 million patients [4]. Worldwide, 537 million individuals suffer from diabetes and this figure is anticipated to rise to 643 million in 10 years and 783 million by 2045, according to the prediction in 2021 [5]. The risk of complications from diabetes can be decreased through its early detection and treatment, which involves maintaining blood sugar levels within a normal range [3].

### 1.2. Obesity: Its Role in Diabetes and Diabetic Retinopathy

Approx. 68% of patients with T2DM are overweight with significant central visceral adiposity (and a body mass index (BMI) ≥ 30 kg/m^2^)) indicating the important role of adipose tissue and obesity in the development of the disorder [5,6,7]. Due to obesity, T2DM has become a global epidemic, the complications of which include retinopathy, nephropathy, and difficult-to-heal ulcers [8]. It is well known that the chronic metabolic changes characteristic of T2D can lead to numerous complications, which are generally grouped into macrovascular and microvascular diseases [5]. The Middle East (11.75–17.72%) and Oceania (15.42–27.25%) regions of the world have the highest diabetes rates due to nutritional and lifestyle errors [8]. The Asia-Pacific is home to half of the people with diabetes in the world; specifically, China and India have achieved recent exponential economic success and adopted dramatic Western-type lifestyle changes, such as unhealthy diets and physical inactivity leading to obesity, high blood pressure (BP), and high cholesterol, alcohol consumption, cardiovascular disease, and smoking, contributing to the rising prevalence of diabetes and DR [9,10]. In addition, obesity has been a risk factor for several systemic diseases, including hypertension, stroke, dyslipidemia, and sleep apnea, and these diseases have been reported as potential risk factors for DR [10]. As a result of diabetes, the number of outpatient and elective operative interventions for complications has also increased, especially for abscesses, difficult-to-heal arterial–venous and decubitus ulcers, diabetes foot, limb amputations, eye surgeries, kidney transplants, etc. [11,12]. 

DR is considered a hyperglycemia-induced secondary vascular and neurodegenerative disease that occurs after several years of poorly controlled hyperglycemia. Inflammation plays a central role in the pathophysiology of diabetes mellitus and metabolic X syndrome, and, in particular, affects innate immunity in the development of complications. Obesity-induced insulin resistance is the consequence of the low-grade chronic inflammation of adipose tissue. The activation of adipose tissue macrophages, together with the activation of nuclear factor-kappa B (NF-κB), leads to the production of several cytokines (interleukins, TNFα). Changes in the retinal vascular endothelium, partly due to premature molecular aging processes, become attractive for the adhesion of leukocytes. The disruption of vascular integrity promotes vascular occlusion and increasing ischemia in the peripheral tissues and retina. These changes can be observed even before significant hyperglycemia [13,14].

The Guangzhou Diabetic Eye Study (2017–2019) and Saudi National Diabetes Registry reported that general and/or centripetal obesity acts as a protective factor in the development of DR more significantly in females compared to males. In conclusion, (abdominal) obesity is a potentially modifiable risk factor of DR. The detection of modifiable risk factors for DR has an important value in terms of the early and intensive treatment of patients with a higher risk of DR, which ensures a better prognosis (See Section 3.5 for further information) [10,13].

### 1.3. Diabetes and Its Impact on the Retina 

Patients with diabetes are at a high risk (approx. 90%) of developing diabetic retinopathy (DR), which is the most common eye-related microvascular complication of DM. DR is currently the leading cause of blindness [3,5]. This can be attributed to the rapidly expanding diabetic population. The UK Prospective Diabetes Study (UKPDS) shows that up to 40% of patients with type 2 diabetics (T2DM) have some degree of DR at the time of diagnosis [15]. Studies confirm that the risk of DR increases in the case of a disease that has existed for more than 5 years, and its incidence is around 17%, while after 15 years it is 97.5% [16]. The mean age of patients when diagnosed with diabetes is 42.5 years old, which is due to high sugar consumption, an unhealthy diet, low physical activity, genetic factors, and lifestyle [17].

The two main forms of DR are (i) proliferative DR and (ii) non-proliferative DR. The latter is the early stage of DR and is also the most common form, causing blurred vision. It can be divided further into mild, moderate, and severe stages based on the extent of the damage to blood vessels in the retina. The former, on the other hand, is a more advanced stage with retinal neovascularization. This vision-threatening form of DR is very serious and can cause macular problems (diabetic macular edema) or lead to retinal detachment due to the formed scar tissue. The diabetic alterations (hyperglycemia, hypoxia, inflammation, etc.) lead to the disruption of the blood–brain barrier and cause excess intraretinal and subretinal fluid accumulation. The formation of the macular edema is responsible for further harmful intracellular processes and the main cause of visual loss [3,9,18,19]. Parallel changes in serum glucose level and visual acuity are described in diabetes. Changes in blood glucose level can cause short-term or permanent refractive changes, which result in myopia or hyperopia [20,21,22]. Hypermetropia is the most common form of ametropia in hyperglycemia patients [23]. 

DR is also categorized as a chronic low-level inflammatory disease. The high blood glucose level promotes the accumulation of advanced glycation end-products (AGEs) and leads to monocyte and macrophage stimulation. In diabetic patients, it has been observed that the levels of proinflammatory cytokines, including monocyte chemoattractant protein-1 (MCP-1), interleukin-6 (IL-6), and tumor necrosis factor alpha (TNFα), as well as matrix metallopeptidase 9 (MMP-9), inducible nitric oxide synthase (iNOS), and the intercellular adhesion molecule-1 (ICAM-1), are elevated in the eye. High levels of serum glucose and the production of mitochondrial and extracellular reactive oxygen species (ROS) can damage endothelial cells and neurons, leading to cell death through apoptosis. The retinal neurons and vascular cells depend on insulin receptor activity, and the reduced insulin action leads to neurodegeneration. Insulin signaling has been shown to have neurotrophic effects on the retina, and a deficiency in neurotrophins may contribute to the development of retinopathy [3,14,24,25,26]. Potential biomarkers, such as nerve growth factor (NGF), apolipoprotein (Apo) A1, lipocalin 1, lactotransferrin, lacritin, lysozyme C, lipophilin A, immunoglobulin lambda (λ) chain, heat shock protein 27 (HSP27), and β2-microglobulin in tears, were identified as being specific to DR, associated either negatively or positively with the condition [5].

At present, it is well-accepted that carbohydrates, protein, fat, vitamins, and minerals are all implicated in regulating miRNA expression. miRNAs are small, non-coding RNAs that can bind to the messenger RNAs (mRNAs) of target genes and post-transcriptionally inhibit their translation or degrade them [27]. Several studies have also shown that miRNAs play a prominent role in DR by modulating multiple pathogenetic pathways, especially apoptosis, inflammatory response, oxidative stress, and neurodegeneration [28,29]. It has also been proved that several miRNAs are differentially expressed in the retina and blood of patients with DR, and they may be novel therapeutic targets for the efficient prevention and treatment of DR [28,30,31,32]. By modulating the levels of specific miRNAs, miRNA therapy can potentially restore the balance of gene expression and correct the underlying causes of diseases [29,33,34]. A miRNA-expression profiling analysis of diabetic rats’ retinas and retinal endothelial cells (RECs) presented differential expressions of miRNAs and revealed miRNA signatures of several pathologic pathways of early DR. In vitro and in vivo studies examined the interactions of miR-146a and nuclear factor kappa B (NFκB) activation, and the role of upregulated vascular endothelial growth factor (VEGF)-inducible miRNAs and the miR-34 family in the pathogenesis of DR [35].

### 1.4. NFκB Signaling

Although VEGF therapy is widely accepted, the NFκB pathway has received less attention in the treatment of DR. NFκB is a redox-sensitive mediator of several different cellular processes and present almost in every cell type. This protein complex has an inactive state, and when different stimuli (such as hyperglycemia, hypoxia, inflammation, etc.) occur, it is activated. During its activation, the IκB (inhibitor of κB) proteins are phosphorylated by IκB kinases in the cytoplasm and then NFκB is translocated to the nucleus. As transcriptional factor, NFκB, regulates the transcription of a wide variety of genes in immune (adaptive, innate) and inflammatory responses, such as pro-inflammatory cytokines, adhesion molecules, and chemokines [36,37,38].

In an aged retina, NFκB shows a higher expression [39] and it is a key mediator of the activation of Müller cells and microglia after different forms of damage or in a high-glucose-level condition [40,41]. Moreover, the intravitreal injection of the NFκB inhibitor (pyrrolidine dithiocarbamate) reduced retinal neovascularization in a mice model of ischemic retinopathy [42]. It showed a higher expression during DR in a rat’s retina compared to the healthy controls. After 4 weeks of diabetes onset, the ganglion cell layer (GCL) and inner nuclear layer (INL) cells showed elevated NFκB expressions and some apoptotic cells in the GCL. Furthermore, the immunohistochemical results also exhibited that NFκB also entered the nucleus at 4 weeks of diabetes. Subsequently, the apoptotic cell number continuously increased and, after 12 weeks, this also occurred in the INL [43]. Ding et al. also described an increased TUNEL-positive cell number in the GCL and higher NFκB expression, VEGF and inflammatory cytokine levels in a diabetic retina. They examined the effectiveness of an intraperitoneal injection of an IKKβ inhibitor (IMD-0354), which reduced the expressions of NFκB, VEGF, inflammatory cytokines, and glial fibrillary acidic protein (GFAP); furthermore, the number of apoptotic cells also decreased in the retina. This treatment also caused the elevation of decreased antioxidants (superoxide dismutase (SOD), catalase (CAT), and glutathione (GSH)-Px) levels in diabetic rat retinas [44]. In another rat DR model, the effectiveness of NFκB inhibitors was also described by reducing ganglion cell death and GFAP expression in N-methyl-D-aspartate (NMDA)-induced retinal excitotoxicity [45]. The intravitreal injection of Resolvin D1 also decreased the degradation of IκB and the phosphorylation of NFκB in streptozotocin (STZ)-induced retinopathy [46]. Elevated NFκB and advanced glycation end-product (AGE) levels were downregulated in diabetic animals after the oral administration of a coconut kernel protein [47]. In an in vitro experiment with a high-glucose-stimulated cell culture of rat Müller cells, curcumin inhibited NFκB translocation to the nucleus; moreover, an intravitreal injection of curcumin suppressed the phosphorylation of NFκB in STZ-treated rats [48].

Since many NFκB targets are important participants of DR signaling pathways and are activated during the early stage of the development of diabetes, its inhibition is presently a promising approach for both conventional and complementary DR therapies.

## 2. Aims

Numerous recent studies have investigated NFκB in the context of ocular surface disorders, including chemical injuries, ultraviolet-radiation-induced injuries, microbial infections, allergic eye diseases, dry eye, pterygium, and corneal graft rejections [49]. As previously mentioned, the main risk factors of DR have a critical role in evolving the retinal inflammation state via oxidative stress, nitric oxide synthase dysregulation, AGE formation, or the inhibition of endogenous anti-inflammatory pathways. The inhibition of key regulator elements (TNFα, NFκB, poly(ADP-ribose) polymerase (PARP), or cyclooxygenase (COX)) in diabetic-induced inflammatory responses is effectively usable for delaying DR-associated vascular complications. 

**Figure 1:**
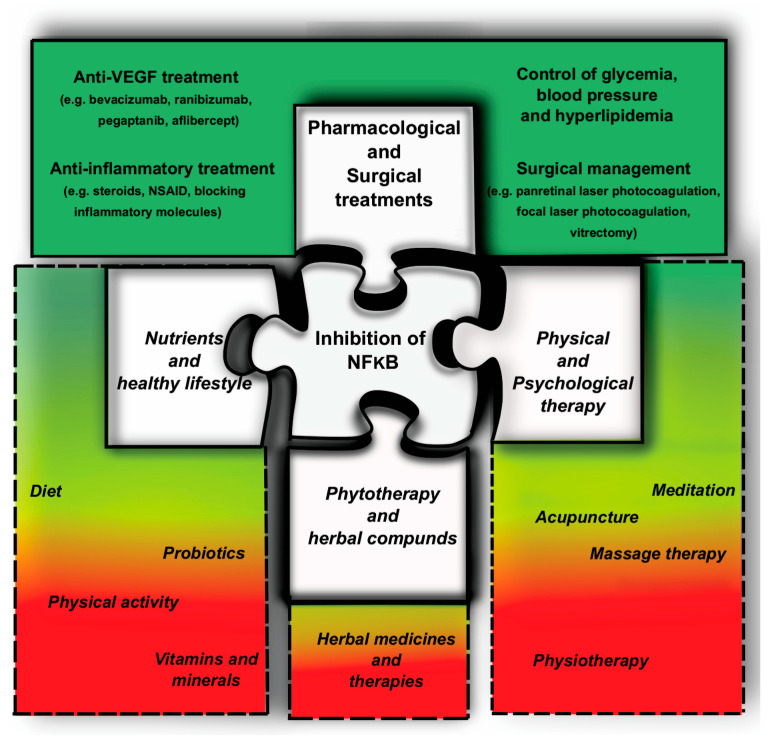
Potential complementary pieces (puzzle pieces with dashed line) of conventional DR treatments (puzzle pieces with solid lines). The change in color shades (yellow to red) indicates the degree of acceptance of different therapies.

The authors present the possible health-preventive methods for reducing the incidence of DR and examine its possible relation to retinal degeneration. These methods include regulating blood sugar levels, reducing psychosocial and oxidative stress, and inhibiting the NFκB signaling pathway, all of which can help decrease the long-term mortality rate associated with diabetes mellitus. Some potential tools for achieving these goals include proper nutrition, herbal remedies, acupuncture, physical exercise, and mind–body practices that can help alleviate oxidative stress (Figure 1). Some of these methods have gained wide acceptance among medical professionals in the past, while others are still in the phase of gaining force by gathering evidence for their usefulness. 

## 3. Management of DR

### 3.1. Evidence-Based Management of DR

The World Health Organization (WHO) [50], the American Academy of Ophthalmology [51], and the Royal College of Ophthalmologists [52] have published guidelines providing evidence-based recommendations for the care of patients with diabetic retinopathy, including screening, diagnosis, and treatment.

The aim is to provide the most effective and appropriate care for each individual patient based on their specific needs and circumstances. The guidelines recommend maintaining good control of blood sugar and blood pressure levels to lower the risk of retinopathy developing or progressing. Screening typically involves a dilated pupil exam to check for vascular changes in the retina. Retinal imaging, such as fundus photography or optical coherence tomography (OCT), may also be used to detect possible pathological signs (such as cotton-wool spots, microangiopathy, etc.). Research was conducted to determine the optimal time between screening tests (also known as the screening interval) for DR. Most studies suggest that the screening interval should be between one and two years [53]. However, some studies have explored the idea of individualizing the screening interval based on individual patient’s risk factors, such as the person’s glycemic control, retinopathy severity, and the cost-effectiveness and affordability of the screening program [54,55,56,57]. A clinical study in Australia showed that patients with poor blood pressure (BP) or lipid control in addition to poor glucose control were at a greater risk of DR, compared with glucose control alone. Good glycemic control is also important as it can lead to the regression of DR. The data from the UK Prospective Diabetes Study [57] and the Diabetes Control and Complications Trial, major clinical trial studies from the United Kingdom and United States involving White populations, showed that controlling blood glucose (HbA1c < 7%) and BP levels slowed the onset and progression of retinopathy [58]. The regular photographic screening of DR is an effective strategy to prevent vision loss from DR. According to the recommendation of the Canadian Diabetes Association (CDA), in the case of T1DM, ophthalmic screening is recommended in patients aged 15 years or older after 5 years following the diagnosis, while in the case of T2DM, it is recommended to perform an ophthalmic screening test when the diagnosis is made [17]. According to the national guidelines, a dilated fundus examination is recommended at least once a year for all diabetic patients, regardless of the presence of ophthalmic complications [59]. The goal is to balance the risks for individual patients with the overall effectiveness of the screening program [60]. Visual impairment caused by DR has a significant negative impact on a patient’s quality of life [61]. In a clinical study in Australia, poor visual acuity from DR in the better eye was associated with decreased functioning and emotional well-being in patients with DR. In a recent study in Australia on patients with DR, poorer self-reported vision in adults with DR was associated with emotional distress through the limitations on daily living activities and social factors [62].

In the last half-century, several proven treatment methods have emerged for ophthalmologists to administer the necessary medication and/or surgical intervention before DR-related vision loss occurs [3,63]. The professional guidance also provides suggestions for the use of medical interventions, such as laser treatment, intravitreal injections, and vitrectomy surgery, to manage the condition [14]. DR depends on the severity of the disease, classified as non-proliferative and proliferative stages. The former refers to early lesions on the eye vasculature, usually without visual symptoms; however, if there is evidence of a macular edema, the vision is diminished. New, abnormal retinal vessels growing relatively rapidly close to the center of the eye comprise the proliferative (second) stage [3]. At present, anti-VEGF inhibitors (e.g., bevacizumab, pegaptanib, ranibizumab, aflibercept, etc.) are the first-line medical options for DME treatment. Several clinical studies have demonstrated the advantage of intravitreal anti-VEGF injections reducing retinal neovascularization in patients with DR [14]. The management of DR in low- and middle-income countries is a major challenge due to lack of access to eye specialists, healthcare resources, and facilities. Xu et al. (2017) reported good clinical efficacy results after using intravitreal ranibizumab and conbercept in 62 Chinese patients over a 12-month clinical study [64]. Japanese patients with diabetic macular edemas were treated with intravitreal ranibizumab and reported predictors of good clinical outcomes, which included better baseline best-corrected visual acuity and a reduced central retinal thickness; hence, this supports the early use of intravitreal anti-vascular endothelial growth factor (anti-VEGF) for DMEs [65]. However, the high cost of treatment, traveling time, and lack of reimbursed healthcare may impair patient compliance; hence, the number of anti-VEGF injections might be suboptimal. 

Adjunct to intravitreal pharmacotherapy, laser photocoagulation techniques (peripheral retinal laser photocoagulation, focal macular laser photocoagulation, and grid photocoagulation) offer a reliable treatment option for proliferative retinopathy, macular oedemas, and DR-associated retinal detachments targeting newly formed and abnormal leaking vessels. In the clinical practice, the pars plana vitrectomy serves as the surgical treatment of vitreous hemorrhages, combined/tractional retinal detachment, macular malformations, and fibrovascular proliferation [14]. Additionally, many patients in both developed and developing countries do not have sufficient resources to cover the long-term treatment costs for DME. As such, ophthalmologists in Asian regions often select focal/grid laser photocoagulation as their mainstay of treatment and patients are only treated when DR has progressed to advanced stages [61]. Others have shown that there is a significant improvement in Japanese patients’ quality of life after vitreous surgery for PDR even without significant corresponding improvements to visual acuity [9].

### 3.2. Possible Future Solutions

At present, the screening and diagnosis of diabetic retinopathy can be supported by cutting-edge technology, such as artificial intelligence (AI) and telemedicine. Retinal scans can be analyzed by AI algorithms to detect DR and avoid blindness [66]. These algorithms have been approved and used for the automatic identification of DR that needs referrals with satisfactory diagnostic results. Telemedicine, which involves the use of digital technology to provide remote healthcare services, has also been used to improve access to DR screening [67]. Screening programs that use telemedicine can allow individuals to capture their own retinal images and send them to screening centers for analysis and further planning [68]. AI is a useful tool for screening and diagnosing DR, but it does not replace regular eye exams and good diabetes care, which are essential to prevent or slow down the onset or worsening of the condition [69].

By restoring the balance of gene expression, miRNA therapy has the potential to address the root causes of diseases. Kovács et al. (2011) conducted the first miRNA-expression profiling analysis of the retina and RECs of streptozotocin-induced diabetic rats (aged 3 months old). At least 86 to 120 (*p* < 0.01) miRNAs were differentially expressed among 350 and 220 miRNAs diabetic rats versus the controls, respectively. A functional annotation revealed the miRNA signatures of several pathologic pathways of early DRs, namely, the upregulation of NFκB—(miR-21, miR-132, miR-146a, miR-146b, miR-155), VEGF-(miR-17-5p, miR-18a, miR-20a, miR-21, miR-31, miR-155), and p53 pathways (miR-34). Furthermore, Tr-iBRB as a conditionally immortalized retinal capillary endothelial cell line was used for in vitro functional assays to examine the dissected interactions of miR-146a and NFκB activation [35]. The research shows that high glucose levels alone do not cause an increase in miR-146a expression in RECs. Instead, these cells respond to cytokines, such as IL-1β and TNFα. This suggests that the increase in miR-146 and other NFκB-responsive miRNAs in diabetes may be due to the paracrine effects of cytokines produced by other cell types in the retina as a result of high glucose levels. An in vivo study also corroborated the pivotal role of miR-146a in NFκB activation pathways. The intravitreal injection of lenti-miR-146a in diabetic rats resulted in an increased expression of miR-146a, while its key downstream target genes (CARD10, IRAK1, and TRAF6) were downregulated and led to the downregulation of the NFκB downstream gene, ICAM1, a proinflammatory factor attracting leukocytes docking on endothelial cells [70]. Increased levels of VEGF-inducible miRNAs, such as those in miR-17 clusters, may play a role in the development of diabetic retinopathy by affecting both leukostasis and angiogenesis [71,72,73]. Members of the miR-34 family may also contribute to the disease by promoting the p53-induced apoptosis of neuroretinal and endothelial cells. This is supported by studies showing that blocking miR-34 can prevent p53-induced apoptosis [74,75]. 

Another study by Santovito et al. (2021) found that DR was associated with higher circulating miR-25-3p and miR-320b levels and lower levels of miR-495-3p in a cohort of patients with T2DM with DR, compared with diabetic subjects without DR and healthy individuals. The circulating levels of these miRNAs correlated with the severity of the disease and showed a high accuracy for identifying DR. The authors suggested that these miRNAs may have served as putative disease biomarkers and highlighted novel molecular targets for improving the care of patients with DR [28,33]. Furthermore, Zampetaki et al. (2021) studied the plasma miRNA signature in patients with T2DM and its association with DR. They found 13 miRNAs that were differentially expressed between T2DM patients with and without DRs and confirmed their findings in a separate group of patients. Their research showed that changes in the levels of miR-15a, miR-29b, miR-126, miR-223, and miR-28-3p occurred before the onset of the disease [76]. One of the miRNAs most strongly linked to diabetes was miR-126, which was abundant in endothelial cells and helped maintain their health and the integrity of blood vessels by suppressing negative regulators of the VEGF pathway, such as SPRED1 and PIK3R2/p85-β [77]. Ruiz et al. (2015) confirmed that miR-200b was upregulated in the retinas of diabetic rats and humans, and it promoted inflammation and vascular leakage by targeting ZO-1, a protein that maintained the integrity of the blood–retinal barrier. Inhibiting miR-200b with an antagomir can reduce inflammation and vascular leakage in diabetic rats [78]. Greco et al. (2020) investigated this to reveal the differences in circulating miRNAs between patients with and without DRs, examine their predictive values, and understand their pathogenic impacts. The study found that miR-1281 was significantly higher in patients with DR and could potentially serve as a biomarker for the condition, providing insights into a new potential target for treating DR [79].

**Figure 2:**
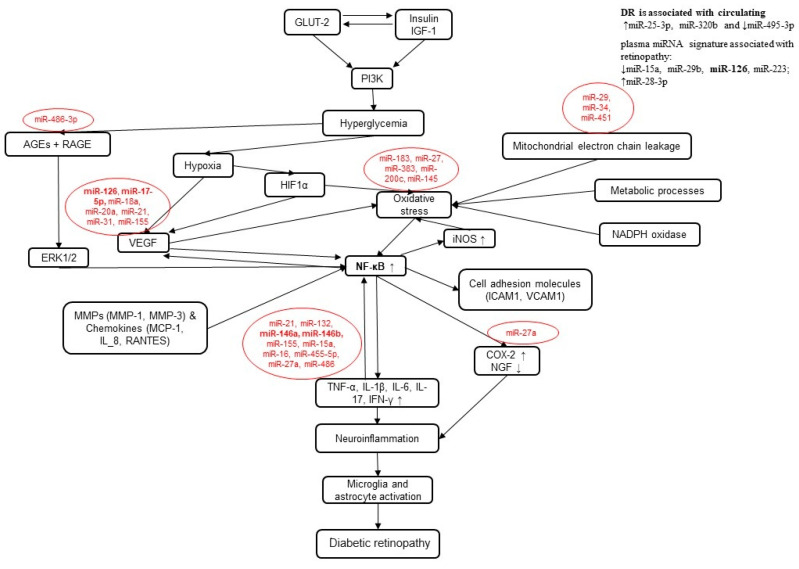
A schematic overview of the interacting pathways involved in the development of diabetic retinopathy centered on the NFκB pathway. Red bubbles contain the miRNAs that can play a pivotal role in targeting certain nodes.

According to Perez-Santos et al. (2020), DR can be treated by the administration of a miR-let-7b inhibitor, which affects the levels and functional activity of miR-let-7b. Targeting miRNA, tailoring miRNA and protein expression, can prove to be a potent and effective treatment strategy for DR [80]. However, new miRNAs involved in complications are being explored and their experimental target verification is still a great concern for researchers. More studies exploring miRNA-based treatment strategies to evaluate their protective and causative functions in diabetes (Figure 2) and its complications are needed.

### 3.3. Relevance of Complementary Medicine

No matter how ophthalmic diseases have major public health importance (irreversible visual impairment and blindness caused by infections, glaucomas, cataracts, DR, and dry eye), unfortunately they are quite neglected in phytotherapy [81]. A limited number of trials on herbal remedies have been published to date in the field of ophthalmology, and almost no research has been conducted on the complementary use of aromatherapy–apitherapy tools [82]. In a Canadian survey of glaucoma patients, 13.7% reported a current or previous use of complementary medicines for their eye conditions, which were not disclosed to the ophthalmologists by nearly two-thirds of the patients. Unfortunately, similar studies have not been published for DR yet. More than 40% of glaucoma patients believed that the complementary treatments helped to improve their condition. Given the widespread use of herbal medicines and the tendency of patients to not inform of their use to their providers (65%), it is important for eyecare professionals to educate their patients on the safety and efficacy of commonly used herbal medicines [81]. Herbal therapies at the same time have serious limitations, or even drawbacks, because many patients do not consider them as medicines and do no inform their doctors about using them. The market for dietary supplements is weakly regulated and there are concerns about quality assurance and misleading marketing. The improper use of medicinal herbs can carry risks, and herb–drug interactions should not be ignored. However, complementary medicine is recognized as an influential factor, with medical schools including it in their curricula and medical societies using it in practice and research. The European Union has established guidelines to protect consumers through the Committee on Herbal Medicinal Products [81,82,83,84]. 

In the past decade, there has been a significant increase in the use of complementary and alternative medicine (CAM) worldwide for managing conditions, like diabetes. The reports indicate that as many as 72.8% of individuals with diabetes may use CAMs [83]. The rising popularity and the trend of more and more products appearing on the market cannot escape the attention of ophthalmologists either, as their patients suffer from cataracts, glaucomas, AMD, and DR. They are also increasingly searching for such medicinal products [84]. However, most of these medicinal products still lack the appropriate evidence and side-effect profiles [82].

### 3.4. Mind–Body Therapies

To prevent DR and other diabetes-related pathologies, healthcare professionals should start their treatment in concordance with the early stage of the disease. The first steps always include physical activity and lifestyle changes. In 1927, Katsuzō Nishi developed the Nishi Health System, a lifestyle program that combines dietary and exercise elements. The program includes vibration warm-up exercises designed to strengthen the muscles in the limbs and improve the capillary microvascular system. At present, the Nishi Health System is practiced by Aikido athletes and visual therapists [83]. Zalmanov created a similar target for intervention in the 1950s as he invented the “turpentine bath” balneotherapy targeted to increase limbal microcirculation. The “capillary theory”, since the source of many diseases was identified by him and his observations, was correct regarding diabetic patients’ abnormal capillary features (e.g., crossing capillary vessels) in their fingers, which was associated with a risk of DR, even Alzheimer’s disease [85,86]. Hyperglycemia-induced AGE/RAGE, ROS, and HbA1c activated apoptosis in the nerve fibers and insulin resistance via the activation of NFκB and the release of TNF-α. NFκB overexpression led to neuronal leukocyte infiltration and decreased NGF, IL-6, IL-1β, and TNF-α. Increased ICAM and NFκB expressions following an inflammatory response were observed in the microvessels of the sciatic–tibial nerves of diabetic rats leading to the narrowing of vessels and ischemic conditions. PPARs are reduced in the nerves in response to increased chemokines that enhance neuronal death [87]. 

Traditional Chinese medicine (TCM) applies Qigong (气功) as a therapeutic tool, a body exercise practice for healing interventions, which uses positions, movements, and breathing exercises combined with meditation to induce vegetative regulation (homeostasis). Breathing is particularly important to achieve vegetative control for muscle tone and capillary flow. The prime benefit is stress reduction, which is believed to be the main source of all civilizational diseases. Infrared thermography demonstrated that frequently performing Qigong exercises can alter acral temperature (37 °C), attributed to increased capillary microcirculation. Considerable numbers of studies also present the beneficial effects of physiological parameters, like blood pressure, heart rate, lung functions, and even serum lipid parameters. Qigong also seems to benefit the overall quality of life of the elderly with chronic diseases, such as DM and hypertension [88,89,90,91].

### 3.5. Physical Activity and Exercise

The correlation between physical inactivity and the onset of diabetes has been established and confirmed to account for almost 27% of all causes of this disease. Physical inactivity is classified as one of the risk factors, not only for the onset of T2DM, and it is also a key contributor to many complications, such as renal failure, nephropathy, and DR [18]. There is strong evidence that losing excess weight can delay the progression of diabetes (from prediabetes to type 2 diabetes). Aerobics and other exercises are regularly recommended to patients. Daily exercise, or at least performing it two times a week, is recommended to reduce insulin resistance, regardless of the type of diabetes [92,93]. Accordingly, the Finnish Diabetes Prevention Study proved that an intensive dietary and physical health program reduced the risk of developing diabetes by nearly 58%. Similarly positive results can be observed in Diabetes Prevention Program studies, where weight reductions in obese individuals with impaired glucose tolerance levels reduce the incidence of DM by approximately 58%, while metformin reduces the incidence of DM by 31%. Based on the studies of the American Cancer Society’s Cancer Prevention Study, a 10 kg weight loss outcome reduced total mortality by 25% in obese patients with diabetes [93].

Physical activity was reported to have beneficial effects on glaucoma, AMD and DR, and exercises, such as jogging and cycling, affecting both IOP and ocular hemodynamic parameters [94]. Szalai et al. applied the term “trained eye”, like the “trained heart”, to emphasize the beneficial effects of physical exercise on the visual system as well. They found that the nonathlete group had significantly reduced vessel densities across many retinal microvascular structures [95]. According to a review by Bryl et al. (2022), physical activity can reduce the risk of developing DR in diabetic patients. The authors also noted that physically less active diabetic patients showed increased blood flow in the retina when they exerted themselves. Physical exercise has therefore been postulated to present a protective effect on retinal health as it has been shown to both lower the risk of AMD and improve visual outcomes [96]. Soleimani et al. (2023) also found that moderate-intensity aerobic exercise significantly decreased fasting blood sugar levels in DR patients [97]. Tikkanen-Dolenc et al. (2017) found that a higher frequency of leisure-time physical activity led to a significant reduction in the risk of severe DR, but there was no association between physical activity intensity or the duration of each session and severe DR [98]. Aro et al. assessed if there was an early incorporation of lifestyle changes in diabetes therapy at the onset; this beneficially influenced the occurrence of DR [99]. Some other studies depicted the positive ophthalmic effects of higher physical activity, including neuro-protectivity through brain-derived neurotrophic factor (BDNF) signaling [100], increasing macular choroidal thickness [101], lowering the risk of AMD [102,103], and decreasing IOP and the need for the retinal photocoagulation treatment of DR [97,104].

A limited number of pilot or clinical studies suggest that massage therapy should be used as a complementary therapy for DR. Massage therapy, including traditional European or Swedish massages, Thai, reflexology, and aromatherapy-supplemented foot massage, has been found to be effective in reducing pain and improving functions in patients with diabetic neuropathies by improving their circulation, releasing muscle tension, and reducing anxiety [105,106]. Essential oils distilled from herbs, such as *Rosmarinus officinalis* L., *Pelargonium* x *asperum* Ehrh. ex. Willd., *Lavandula angustifolia* L., *Eucalyptus globulus* Labyll., and *Matricaria recutita* Rauschert, have been shown to relieve neuropathic pain [107,108]. In India, Ayurvedic oil reflexology foot massages (Padabhyanga) have a long tradition of being used as a supplement to ophthalmic therapies, although thier effectiveness has not been proven through evidence-based research [109]. 

Novel eye and body exercises were developed and researched by Kolpakov et al. They developed a complex of gymnastics and massage therapies, which could improve eye health, which was approved by the USSR Ministry of Health in 1987. It was intended for improving the state of the cardiovascular system, relieving fatigue, and increasing efficiency, presenting a beneficial effect on the blood supply to the eyes. Kolpakov and Kogan patented (Patent No: RU2105534C1) their invented exercise program performing general gymnastics and self-massages [110,111,112,113]. The exercises termed “industrial” (workplace) and “hygienic” (home) gymnastics are part of the official education and examination requirement for registered visual therapists. Another Russian patent (Patent No. RU2612596C1) was invented by Komarova and Belenko as a method for the improvement of retinal blood circulation in patients with various ocular pathologies and postoperative conditions [114]. Both Kolpakov and Komarova suggested that their physical and eye exercises were ideal for AMD and DR patients [111,112,113,114]. The exact mechanism occurring during or after exercises that provided both benefits for and the protection of the retina remain unknown [18]. It is postulated that exercise can precondition individuals to become less susceptible to chronic inflammation. The molecular effects include the inhibition of inflammatory responses and oxidative stress; so, prolonged daily sedentary behavior is closely associated with a higher incidence of retinal dysfunction in diabetic patients [18]. Liu et al. (2018) concluded that moderate exercise training decreased NFκB activation and repressed pro-inflammatory gene (IL-6 and TNFα) expression in the skeletal muscles of diabetic mice. In T2DM, lifestyle modifications, such as moderate exercise, may help maintain muscle health [115]. In obesity and T2DM, adipose tissue GLUT4 expression is lower than that in age-matched, healthy subjects. Exercise training has been shown to increase adipose tissue GLUT4 content and GLUT4 mRNA in the skeletal muscles of T2DM patients. Exercise training remains the most potent stimulus to increase skeletal muscle GLUT4 expression contributing to improve insulin action and glucose disposal and enhanced muscle glycogen storage following exercise training in health and disease studies [116]. 

Possible side effects and limitations: it is important that proliferative DR and high myopia tend to vitreous liquefaction, retinal and vitreous bleeding, vitreous-induced retinal tear formation, or retinal detachment. In various types of vitreous-induced retinal tear formations, it is thought that vitreous movement causes the spontaneous rupture of small retinal capillaries. Similar, harmful ocular effects can occur from vibratory and sling-shot sources, like bungee jumping, which may produce an exaggerated vitreoretinal traction effect [117,118]. Due to the abovementioned risks, the authors did not recommend high-intensity Nishi, Kolpakov, and Bates body-swinging exercises to avoid vitreoretinal injuries in advanced-stage proliferative DR, including highly myopic eyes with known peripheral retinal pathologies.

### 3.6. Phytotherapy and Herbal Compounds

Worldwide, many medicinal herbs or herbal extracts are used to treat diabetes and DR. A variety of herbs, including *Momordica charantia* L., *Trigonella foenum-graecum* L., *Gymnema sylvestre* R. Br., *Azadirachta indica* A. Juss., *Zingiber officinale* Roscoe, *Sesamum indicum* L. (oil), *Allium sativum* L., *Andrographis aniculate* (Burm.f.) Nees, and *Aloe vera* (L.) Burm.f., have been used to manage diabetes. These herbs have antidiabetic properties, such as increasing insulin secretion, promoting glycogenesis and hepatic glycolysis, mimicking adrenaline, blocking pancreatic beta-cell potassium channels, activating cAMP, and regulating glucose absorption in the intestines [84]. Wickramasinghe et al. (2021) presented a review article enumerating herbs (*Artemisia dracunculus* L., *Centaurium erythraea* Rafn, *Cornus officinalis* Sieb. et Zucc., *Gynura divaricata* (L.) DC, *Hibiscus rosa sinensis* L., *Lactarius deterrimus* L., *Myrica rubra* Sieb. and Zucc., *Panax ginseng* C.A. Meyer, *Tamarindus indica* L., *Teucrium polium* L., *Thymus praecox* subsp. skorpilii var. skorpilii, *Uncaria tomentosa* (Willd.) DC, and *Woodfordia fruticosa* (L.) Kurz) targeting improved pancreas β-cell functions and β-cell regeneration for the management of DM [119]. *Achillea millefolium* L. was originally used for wounds, minor hemorrhages, skin diseases, and dysmenorrhea in global indication studies. Moreover, South American ethnopharmacobotany documents its antidiabetic and anti-hypercholesterinemic effects [120]. The hydroalcoholic extract of the plant presented an in vivo antidiabetic effect through α-glucosidase and lipase inhibition, insulin secretion enhancer, and potential insulin sensitizer through PPARγ/GLUT4 overexpression stimulation [121]. Many studies have found that the herbs previously mentioned could interact with antidiabetic medications, often resulting in an enhanced effect. For example, when combined with aloe, medications, like glibenclamide, pioglitazone, or repaglinide, had a stronger anti-hyperglycemic effect than aloe alone. These combinations of herbs and medications have been shown to have antidiabetic properties that affect both insulin-dependent and insulin-independent pathways [84]. 

Herbal formulas and herbal extracts may also exhibit various mechanisms to preserve the retina from DR and eventually glaucomatous neurodegeneration [14]. Polyphenolic plant compounds (curcumin, apigenin, quercetin, (E)-cinnamaldehyde and (E)-resveratrol, silymarin, pathenolode, ergolide, andalusol, kamebanin, kamebacetal A, kamebakaurin, excisanin A, hypoestoxide, helenalin, pristimerin, epigallocatechin gallate, avicin, capsaicin, and oleandrin) usually exhibit anti-inflammatory effects in cell culture studies [122,123,124]. Pharmacophytones often interact with anti-apoptotic pathways and inhibit proapoptotic pathways (e.g., NFκB and caspase activity), thereby preventing apoptosis and autophagy. Many of them share the same molecular target: arachidonic acid-dependent and -independent pathways (both signaling through NFκB). Polyphenols may inhibit kinases by inhibiting their phosphorylation or ubiquitination and preventing the subsequent degradation of IkB. This process prevents NFκB’s translocation to the nucleus and the transcription of pro-inflammatory cytokines [125]. 

Traditional Chinese (TCMs), Tibetan (TTMs), and Mongolian medicines (TMMs) have been established by ancient Chinese medical scientists and physicians based on extensive experimental clinical practices. Based on the syndrome differentiation in TCMs, DR is mainly characterized by the deficiencies of Qi and Yin. The characteristics of multiple active compounds and multi-targets determine that TCM can mediate various biological processes in the treatment of DR. The prescriptions of TCMs or herbs have considerable advantages in the treatment of DR, with the primary functions of clearing heat, promoting blood circulation, removing blood stasis, and replenishing Qi. These traditional medical disciplines are abundant with herbal medicines. According to the Chinese saying, “food and medicine have the same root” (食药同源), TCM ophthalmology also includes dietary recommendations for patients. As an example, the main active ingredients of *Lycium barbarum* L. (goji berry, 枸杞) are *Lycium barbarum* polysaccharides (LBPs), which are water-soluble glucoconjugates (rhamnose, xylose, glucose, mannose, arabinose, and galactose). Based on its main pharmacological effects, it is antioxidant, neuroprotective, immunomodulatory, and VEGF-inhibiting, so it is important to the complementary treatment of eye diseases. It has already been investigated in studies in connection with glaucoma, AMD, DR, and retinitis pigmentosa [14,126]. Chiosi et al. examined the Intravit^®^ (OFFHEALTH Spa, Firenze, Italy) supplement containing natural molecules, like curcumin, artemisia, bromelain, and piperine, which can decelerate the advancements of DR, diabetic macular edema, and neovascularization [127]. Baicalein is an isolated flavonoid from the roots of *Scutellaria baicalensis* Georgi, which has been used for centuries as a folk medicine in China and Japan, for the treatment of inflammatory diseases, preventing the secretion of inflammatory and/or cytotoxic factors, consequently protecting neurons and vasculature from damage in DR [14,128]. Astragalin, a 3-*O*-glucoside extracted from *Astragalus membranaceus* Bunge and *Astragalus propinquus* Bunge, has a history of use as a herbal medicine in TCM [14,128]. A root-derived polysaccharide of *Astragalus membranaceus* was found to attenuate high glucose-induced oxidative stress, mitochondrial damage, endoplasmic reticulum stress, and cell apoptosis by suppressing miR-195 and miR-204 expressions in RPE, pointing toward a complex action, interfering at different levels of signal transduction. The bark of *Phellodendron chinense* C.K.Schneid. is mostly recommended to be included in the medical formulas of TMM for its beneficial effects on the eyes and its microcirculation. The prescription of Siwei Jianghuang decoction (四味姜黄汤散, TMM name: Xieriga-4) exhibited beneficial effects on DR via the combined synergistic action of multiple formula components, including 25 g of Curcumae Rhizoma, 25 g of Tribuli Fructus, 20 g of Gardeniae Fructus, and 15 g of Phellodendri Cortex. The herbal treatment significantly reduced the levels of blood glucose and normalized renal serum parameters. It downregulated serum TGF-β1 levels and the expressions of HIF-1α, VEGF, and TGF-β1 at both mRNA and protein levels. The Siwei Jianghuang decoction combined with two other Mongolian herbal formulations (Sugmul-10 and Narenmandula-11) prevented or ameliorated DR through regulating MMP-2 and TGF-β1, the former one being an unusual target of herbal medicines [14,129,130]. 

*Rosa damascena* Mill. hydrosol contains citronellol, 2-phenylethanol, and geraniol as major substituents and other monoterpene alcohols, such as linalool, α-terpineol, 4-terpineol, and nerol. By inhibiting the key enzyme in the polyol pathway, *R. damascena* has the potential to be a natural protector against diabetic cataracts; however, further studies are essential to ensure the efficacy in the extrapolation to human diabetic patients [131]. It has been used in Ayurvedic treatment for eye diseases. Oral consumption causes no toxic side effects on hematologic, renal, and hepatic functions. It also inhibits lipid peroxidation in vitro and has an antioxidant effect similar to tocopherol; thus, it can be beneficial for the prevention of DR by reducing the number of free radicals [131,132]. Similarly, *Cinnamomum* spp. (like *C. verum* J. Presl and *C. cassia* (L.) J. Presl) is a widely popular component of antidiabetic herbal supplements due to its protection against long-term hyperglycemia-induced retinal abnormalities, mainly through its hypoglycemic properties [133].

Recently, a few experimental studies have been conducted on the hypoglycemic effect of *Juglans regia* L. leaf extract in DM. These studies documented that, after the consumption of 100 mg of *J. regia* leaf extract for 3 months and 200 mg for 2 months, the administration of *J. regia* leaf extract significantly reduced blood sugar levels and glycated hemoglobin (HbA1c) compared to the placebo control groups. The *J. regia* leaf extract inhibited protein tyrosine phosphatase 1B (PTP1B) and enhanced glucose uptake. The lipid peroxidation level and catalase also improved significantly after its consumption. The attenuation of caspase-3, COX-2, PARP, and S100B allowed for the detection of the amelioration of retinal degeneration in *J. regia* leaf extract-treated rats in the experiment of Nasiry et al. Walnut leaf extract was considered as protective against DR [134,135,136].

In Indian Ayurvedic medicine, a formulation called *Triphala churna*, which is a powdered fruit mixture of *Terminalia bellirica* (Gaertn.) Roxb., *Terminalia chebula* Retz., and *Phyllanthus emblica* L. in equal proportions, is used to treat diabetes, obesity, wounds, and ulcers. Its flavonoids, tannins, and polyphenols have anti-inflammatory and antioxidant properties inhibiting aldose reductase and sorbitol dehydrogenase pathways, alleviating the cataractogenic and retinopathic complications of diabetes [137]. This formula used in Ayurvedic medicine is also a suggested “add-on” therapy for the treatment protocol of DR to complete the evidence-based therapy for better results [138]. 

Troxerutin is a hydroxyethylrutoside flavonoid, derived from *Styphnolobium japonicum* (L.) Schott, characterized by its free radical scavenging ability, and has been shown to reduce neovascularization and VEGF protein production in the retinas of diabetic rats. Moreover, in humans, one study showed that troxerutin administered at high doses effectively counteracted retinal vein occlusions [139]. 

The precautions and limitations concerning herbal medicines are also worth mentioning. In the first place, *Hypericum perforatum* L. presents several pharmacological interactions by cytochrome P450 induction [140]. Interactions with antiglaucomatous β-blocker medications can result in the failure of achieving the optimal IOP levels [14]. In DR patients, there are several antidiabetics (e.g., tolbutamide and gliclazide) that can cause side effects or lead to therapeutic ineffectiveness [141]. We recommend regular medical monitoring when DM patients consume herbal medications with antidiabetics at the same time.

### 3.7. Nutrition Supplementation

Drug-based therapies tend to be monotherapies with a single drug for a single target; however, the nutritional deficiencies are often multifactorial. Nutrients and nutraceuticals with antioxidant, anti-inflammatory, or anti-apoptotic properties have been extensively studied for their neuroprotective effects [14,141]. In our previous review, we also highlighted the importance of traditional Chinese–Tibetan medicines in DR, where we refer to the formula of herbal compounds in the Supplementary Materials [14].

Micronutrients, such as vitamins, minerals, and phytonutrients, have essential roles in the homeostasis of the retina. Moreover, any deficiencies in their expressions are associated with several retinal diseases. Multiple studies have shown that vitamins A, B1, folate, B12, C, D, and lutein can improve endothelial function, protect neurons, lower blood pressure, and improve visual acuity in DR [141,142]. 

Vitamin A is a precursor of rhodopsin synthesis. Additionally, its deficiency lowers the maintenance levels of NGF and BDNF, which ordinarily protect the retina from oxidative stress injury. Replenishing vitamin A restores NGF and BDNF levels in the brain and retina. Previous studies have shown that the serum levels of retinoids are lower in diabetic animals [143]; moreover, a single intraperitoneal injection of retinal 9-cis can improve visual function by reducing apoptosis and oxidative stress in DR [144]. Zhang et al. described the connection between a lower risk of DR and higher dietary intake of retinal 9-cis [145]. 

B vitamins (B1, B2, B3, B5, B6, folate, and B12) are critical for neural, vascular, and visual functions, so their insufficiencies are the root cause of many chronic illnesses as causative or aggravating conditions. In diabetic patients, vitamin B serum concentrations are lower, possibly due to higher renal clearance and lower reabsorption levels. A vitamin B12-supplemented diet prevented retinal thinning, apoptosis, diabetic gliosis, and overexpressions of VEGF and HIF1α in rats with DR [146]. The plasma levels of vitamins B1, B6, B12, and folic acid were lower in diabetic patients compared to the controls [147,148].

Water-soluble vitamin C (ascorbic acid) is essential for regenerating other antioxidants, such as vitamin E and glutathione [149]. Its oral administration lowers blood pressure levels in patients with essential hypertension, reducing capillary endothelial dysfunction [150]. Patients with a proliferative DR have a 10-fold-lower vitreous ascorbate concentration and an increased tendency to develop a diabetic macular edema [142]. Vitamin C consumption with statins reduces non-proliferative DR in a dose-dependent way, better than statins alone [151]. In a study on diabetic participants, a lower risk of DR was associated with higher vitamin C levels in the serum; furthermore, DR patients had lower vitamin C levels compared to the controls [152]. 

Fat-soluble vitamin D (or “D hormone”) consists of a mixture of vitamin D2 (ergocalciferol) and D3 (cholecalciferol) [153]. The known sources of the vitamin are cereals, mushrooms, various fish (salmon, sardine, herring, mackerel, cod liver oil, etc.), egg yolk, red meat, and liver. Sunlight exposure should also be mentioned as an important inorganic resource [154]. Low-serum vitamin D levels in patients with DM are associated with a higher risk and severity of DR. Since vitamin D supplementation reduces intracellular ROS decreasing VEGF expression, it is beneficial for reducing the risk and severity of DR. Vitamin E supplementation quenches free radicals and reduces retinal oxidative stress levels in the retina, and a daily consumption of 1800 IU of vitamin D improves retinal blood flow. Low-serum zinc levels are correlated with the duration of diabetes, elevated HbA1c, hypertension, and microvascular complications [142]. Intramuscular treatment with vitamin D is helpful in preserving retinal thickness and the GCL cell number; moreover, it inhibits apoptosis in the retinas of diabetic rats [155]. 

We should also mention the roles of generally antioxidant carotenoids. Lutein and zeaxanthin cannot be synthesized in the human body, but they are essential in assisting with retinal phototransduction. Once consumed with food, they easily cross the blood–brain and blood–retina barriers. They are believed to protect against AMD and DR [128]. Astaxanthin is present in algae (e.g., *Haematococcus pluvialis*, *Chlorella zofingiensis*, and *Xanthophyllomyces dendrorhous*), plants, and seafood. This compound inhibits neurotoxicity induced by ROS and its oral intake reduces total hydroperoxide levels in aqueous humor. It inhibits NFκB activity and thereby attenuates RGC’s apoptosis. With the downregulation of mTOR complexes (the AMPK-mTOR signaling pathway that may regulate autophagy). Under hyperglycemic or hypoxic conditions, astaxanthin suppressed VEGF expression by downregulating HIF1α, ICAM-1, IL-6, MCP-1, and XBP1, and therefore CNV in the retina of diabetic rats. It retained the structural integrity of RPE cells by protecting the tight-junction protein (ZO-1) and reduced hypoxia-induced RPE permeability. Administrating astaxanthin as a nutrient is safe and might cause increased bowel movements and a red stool color. It can serve as a beneficial nutritional supplement for the treatments of DR, AMD, and glaucoma [156,157].

We conclude that diabetic patients at risk of developing DR may benefit from dietary supplementations.

## 4. Discussion

The prevalence of DR has increased in the working population and is the leading cause of blindness worldwide; however, to date, there are no effective treatments to reverse its severe neural and vascular outcomes. CAMs are presently receiving more attention, especially natural products, which are the most commonly used complementary options [158]. In the USA, people spend more than USD 30 billion per year on CAM therapies [159,160], while 80 percent of the populations in Africa and Asia use natural drugs for different pathologies [161]. In the present review, we summarize our knowledge about the available complementary therapies for DR treatments, focusing on NFƘB signaling, which plays a central role as a promising target in DR research studies. 

### Risk and Benefits

CAM users are usually patients with chronic conditions who try to ameliorate their symptoms (such as chronic pain and fatigue) with complementary approaches added to their standard medical care. Unfortunately, these chronic patients easily become the targets of overly eager promises and marketing strategies because of their vulnerability. Moreover, they commonly only collect information from the Internet or from their closer circle of acquaintances, family members, and they do not inform their practitioners about their chosen complementary therapies [162,163]. In some cases, this can modify the effect of conventional medicines through the interaction between therapeutic agents when patients use these simultaneously [163,164]. Furthermore, users usually think herbal therapies do not present adverse effects or they cannot be overused; although, they can also present side effects and reference doses. The use of these medications always requires caution (quality, frequency of use, etc.) and patients must discuss the use of these medications with professionals who have a full understanding of their medical conditions [163]. From the technical side, their validation, poor standardization, possible toxic elements, and quality control also comprise the limitations of herbal drugs.

CAMs offer several different types of alternatives for improving health issues in ophthalmology. In the conventional treatment of DR, in addition to surgical management and anti-VEGF therapy, other main drug groups in DR treatment exist, such as AGE inhibitors, PKC-β inhibitors, non-steroidal anti-inflammatory drugs, antioxidant compounds, renin angiotensin–aldosterone system blockers, aldose reductase inhibitors, and angiopoietin 2 inhibitors [165]. These medicines usually target a single, well-known participant of the molecular mechanism. In contrast, herbal medicines and supplements can have multiple targets. Nutritional supplements also target the DR cellular pathology at several main nodes, such as oxidative stress [166], angiogenesis [167], inflammation [168], apoptosis, and autophagy [146]. On the other hand, they have more attack points in the progression of multifactorial diseases, but reduced side effects compared to synthetic medications. Non-pharmacological, mind–body therapies focus on the connection between the mind and the whole body. These are helpful for stress/pain management and improve one’s quality of life, and also offer multi-faceted solutions for multiplex diseases management purposes. There are some important limitations where both the technical aspects (validation, poor standardization, and quality control) and professional medical care sometimes have limited preparation for these new complementary therapies [169].

## 5. Conclusions

In conclusion, our review highlighted the importance of complementary therapies for DR treatments in addition to conventional medicines. We focused on a common molecular target: NFκB. DR is a multifactorial disease with a complex pathological mechanism where, in general, conventional medicines target only one metabolic node of the signaling while complementary medicines often have multiple targets and fewer side effects than synthetic medicines. However, there are some important limitations on the technical side (validation, poor standardization, and quality control), which are important limitations to their application and require further improvements. The use of CAM always requires extra caution (quality and frequency of use) because they can also have side effects or modify the effects of conventional medicines when used simultaneously. On the other hand, physicians are sometimes not up to date with the new, complementary therapies available. In the present work, we underlined how conventional and complementary therapies could work together to achieve a common goal for DR treatment by limiting NFƘB signaling. Overall, our work also points toward the need of further research in this area to minimize the risks and recognize additional benefits. The goal is to find new, promising, overlapping targets in DR therapies similar to NFκB.

## Data Availability

No new data were created or analyzed in this study. Data sharing is not applicable to this article.
